# Hormonal Replacement Treatment for Frozen-Thawed Embryo Transfer With or Without GnRH Agonist Pretreatment: A Retrospective Cohort Study Stratified by Times of Embryo Implantation Failures

**DOI:** 10.3389/fendo.2022.803471

**Published:** 2022-02-03

**Authors:** Leizhen Xia, Lifeng Tian, Shanshan Zhang, Jialyu Huang, Qiongfang Wu

**Affiliations:** ^1^ Reproductive Medicine Center, Jiangxi Maternal and Child Health Hospital Affiliated to Nanchang University, Nanchang, China; ^2^ Columbia College of Art and Science, The George Washington University, Washington, DC, United States

**Keywords:** frozen-thawed embryo transfer, endometrium preparation, hormonal replacement treatment, GnRH agonist, embryo implantation failure

## Abstract

**Objective:**

To evaluate the efficacy of the long-acting gonadotropin-releasing hormone agonist (GnRH-a) administration before hormone replacement treatment for frozen-thawed embryo transfer in women with different times of embryo implantation failures.

**Methods:**

A retrospective cohort study was performed between January 2015 and December 2019. A total of 9263 women who underwent frozen-thawed embryo transfer were included in the study. The study is divided into three parts based on the times of embryo implantation failures. The sample sizes were 4611 for no implantation failure, 3565 for one failure and 1087 for multiple failures. Two endometrium preparation protocols, HRT and HRT with GnRH-a pretreatment (G-HRT), were compared. Confounding factors were treated by propensity score matching and generalized estimation equation.

**Results:**

For women with no failure of embryo implantation, the live birth rate was not statistically different when they underwent HRT and G-HRT (HRT: 42.75% [498/1165], G-HRT: 45.24% [527/1165], P=0.2261). Similar outcome also appeared in women with one failure of embryo implantation (HRT: 47.22% [535/1133], G-HRT: 50.31% [570/1131], P=0.1413). For women with multiple failures of embryo implantation, the live birth rate was significantly difference (HRT: 38.74% [117/302], G-HRT: 45.48% [357/785], P=0.0449). When stratified by age, the live birth rate is similar for women older than 37 years. Generalized estimation equation showed that GnRH agonist pretreatment was independently associated with the live birth rate for women with multiple failures (adjust OR: 1.5, 95%CI: [1.12-2.00]).

**Conclusion:**

For women with no/one failure of embryo implantation, the live birth rate is similar between HRT and G-HRT protocols. For women with multiple failure of embryo implantation, GnRH agonist pretreatment is beneficial to raise the live birth rate.

## Introduction

Frozen-thawed embryo transfer cycles (FETs) have been an important component in the assisted reproduction technology field. The Society for Assisted Reproductive Technology (SART) reported an 82.5% increase in the number of FETs, whereas the number of fresh cycles increased only by 3.1% (1). The rapid increase is mainly attributed to the recent policy of limiting the number of transferred embryos in the fresh cycle, the role of freezing-all embryo strategy in prevention of ovarian hyperstimulation syndrome (OHSS) and the maturation of vitrification freezing technology (2). Also, studies showed that the transfer of frozen embryos resulted in higher rates of live birth than the transfer of fresh embryos, especially for hyper-responders (3).

Endometrial preparation protocol is an important factor affecting the pregnancy rate of FET, and can mainly be divided into hormonal replacement treatment cycles (HRTs) and natural cycles (NCs). Hormone replacement cycle is usually recommended for women with irregular, infrequent menstrual cycles, and can also be utilized in normal ovulatory women because of the flexible time schedule (4). For HRTs, gonadotropin-releasing hormone agonists (GnRH-a) can be used to suppress any hormone production by the ovaries which may interfere with the treatment. However, the efficacy of GnRH-a has been controversial: the latest review showed HRT alone has a lower live birth rate with a low-quality evidence (OR 0.1, 95% CI 0.04 to 0.30, 1 RCT, n = 75) than HRT with GnRH-a pretreatment and a similar clinical pregnancy rate (OR 0.9, 95% CI 0.65 to 1.25, 6 RCT, n = 872) (5). The limited efficacy, adverse effects and expensive cost of GnRH-a seem to suggest its impracticality. However, the women included in the above studies did not limit the characteristics of infertility, such as endometriosis, recurrent spontaneous abortion, repeated implantation failure (RIF). Several studies have shown that GnRH-a suppression significantly enhanced the chances of pregnancy for women with endometriosis or adenomyosis in both fresh and frozen cycles (6, 7). The mechanism is unclear, but studies have speculated that GnRH-a can improve the endometrial receptivity of these women (8).

RIF is the current hot topic. The reasons for RIF could be summarized as uterine/endometrial factors and gamete/embryo factors. Multiple high-quality embryo transfer failures suggested that uterine/endometrial factors were the primarily cause of RIF (9). The speculation of this study was that GnRH-a pretreatment could improve the endometrial receptivity of RIF, and more failures of embryo implantation were associated with lower endometrial receptivity. Therefore, we designed a large sample retrospective cohort study stratified by the times of embryo implantation failures to provide a quantitative reference standard of the use of GnRH-a for doctors.

## Materials and Methods

### Study Design and Population

**Figure 1:**
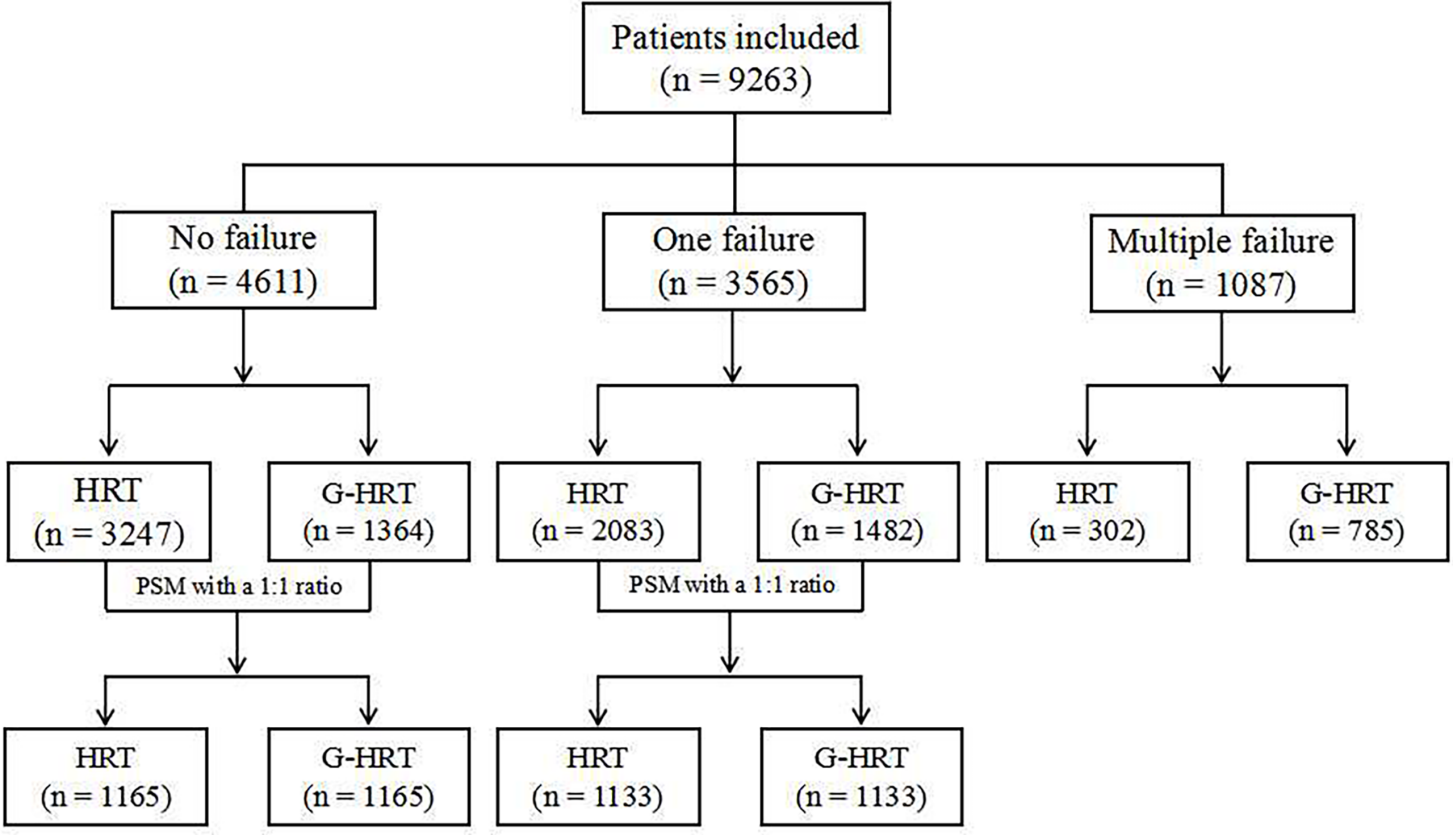
Flow chart of the study.

In this retrospective cohort study, medical records were reviewed for women who underwent frozen embryo transfer treatment between January 2015 and December 2019 in the Reproductive Medicine Center of Jiangxi Maternal and Child Health Hospital Affiliated to Nanchang University in the People’s Republic of China. A total of 9263 women were included in the study. However, 5683 women were actually compared after propensity score matching (PSM). The study is divided into three parts based on the times of embryo implantation failures. PSM method was used to balance the confounders for women with no/one implantation failure, but not used among women with multiple failures, given the similar baseline characteristics between the two groups (**Figure 1**). All women underwent HRT for frozen-thawed embryo transfer with or without GnRH agonist pretreatment. The exclusion criteria includes: (1) endometriosis; (2) adenomyosis; (3) polycystic ovary syndrome; (4) endometritis; (5)intrauterine adhesions; (6) uterine malformation; (7) untreated hydrosalpinx. The study protocol was approved by the Institutional Review Board of Jiangxi Maternal and Child Health Hospital (Nanchang, China).

### Preparation of the Endometrium

#### Hormonal Replacement Treatment (HRT)

Oral estradiol (Progynova^®^; BayerSchering Pharma AG, Berlin, Germany) was started on day 2 or 3 of the menstrual cycle at a dose of 2 mg twice daily. After 6 or 7 days, the dosage of estradiol was adjusted according to the endometrium thickness. When oral estradiol is ≥12d and the thickness of endometrium is ≥8mm, intramuscular progesterone at a dose of 80 mg per day was added. After the endometrial transformation, frozen-thawed embryo transfer was scheduled at 4 days for cleavage-stage embryos and 6 days for blastocyst-stage embryos.

#### Hormonal Replacement Treatment With GnRH Agonist Pretreatment (G-HRT)

A long-acting GnRH agonist (Diphereline, Beaufour Ipsen, France) was injected on day 2 or 3 of the menstrual cycle. The women returned to hospital 28 days later and underwent HRT the same as above.

### Outcome Assessment

The primary outcome was the live birth per transfer cycle, which was defined as delivery of any viable infant at 28 weeks or more of gestation during the first embryo transfer cycle. The secondary outcomes were endometrial thickness, biochemical pregnancy, clinical pregnancy, implantation rate and pregnancy loss. The Serum β-hCG level was measured at 10-12 days after the embryo transfer. A biochemical pregnancy was defined as the serum β-hCG level exceeds 5IU/L, indicating a positive result. Clinical pregnancy was defined as the presence of one or more gestational sacs in the uterine cavity at 30 days after embryo transfer, as detected on transvaginal ultrasonography. Implantation rate was defined as the number of gestational sacs observed on the ultrasound compared with the number of embryos transferred. Pregnancy loss was defined as pregnancies that eventuate in a spontaneous abortion or therapeutic abortion that occurred throughout pregnancy.

### Statistical Analysis

PSM was used to adjust for potential non-similarities between HRT and G-HRT groups. A propensity score was calculated by performing multivariate stepwise logistic regression with age, days of embryo freezing, body mass index, proportion of tubal factors, and number and phase of embryos transferred. The nearest neighbor match without replacement was used in PSM with an 1:1 ratio. In addition to PSM, generalized estimating equations (GEE) based on logistic regression models was also performed to control the influence of confounding factors. The application of GEE mainly considers the clustered nature of data (some patients contributed more than one cycle) (10). Categorical data was described by frequency and percentage. Chi-square test was used to compare the differences between the study groups. Continuous data that conforms to a normal or approximate normal distribution was described as means (± SD) and compared by independent t test. Statistical analysis was tested on two-sided settings, with p < 0.05 considered as statistically significant. All statistical analysis was carried out by SAS version 9.4.

## Results

Baseline characteristics of women with no failure of embryo implantation are presented in **Table 1**. Age, BMI, previous conception, scar uterus, tubal factors, number and phase of embryos transferred were significantly different between HRT and G-HRT group (P< 0.05) before PSM. Age, days of embryo freezing, BMI, previous conception, scar uterus, number and phase of embryos transferred were different between two group (P< 0.05) for women with one failure of embryo implantation before PSM (**Table 2**). After PSM, the baseline characteristics of women with no/one failure of embryo implantation were similar between the two groups (**Tables 1**, **2**). Baseline characteristics were similar between HRT and G-HRT group among women experienced multiple failures without PSM (**Table 3**).

**Table 1:** Baseline characteristics of patients with no failure of embryo implantation before and after PSM.

	Before matching			After matching		
	HRT	G-HRT	P	HRT	G-HRT	P
Patients	3247	1364		1165	1165	
Age (y)	30.64±5	34.14±5.77	<.0001	33.02±5.2	33.21±5.61	0.3803
Days of embryo freezing	402.07±537.64	372.66±472.38	0.0643	399.57±518.35	389.95±484.44	0.6434
BMI (kg/m2)	21.49±2.58	21.98±2.68	<.0001	21.85±2.76	21.75±2.51	0.363
Previous conception (%)	1934 (59.56)	961 (70.45)	<.0001	766 (65.75)	787 (67.55)	0.3561
Scar uterus (%)	567 (17.46)	286 (20.97)	0.0051	247 (21.2)	231 (19.83)	0.4117
Infertility factors						
Tubal factors (%)	2442 (75.21)	970 (71.11)	0.0038	840 (72.1)	860 (73.82)	0.3509
Ovulation obstacle (%)	210 (6.47)	84 (6.16)	0.6949	73 (6.27)	75 (6.44)	0.8651
Male factor (%)	883 (27.19)	339 (24.85)	0.1002	309 (26.52)	285 (24.46)	0.2539
No. of embryos transferred (%)			<.0001			0.2396
1	769 (23.68)	419 (30.72)		362 (31.07)	336 (28.84)	
2	2478 (76.32)	945 (69.28)		803 (68.93)	829 (71.16)	
Phase of embryo transferred (%)			<.0001			0.0676
Cleavage embryo	2451 (75.49)	949 (69.57)		777 (66.7)	818 (70.21)	
Blastocyst	796 (24.51)	415 (30.43)		388 (33.3)	347 (29.79)	

**Table 2:** Baseline characteristics of patients with one failure of embryo implantation before and after PSM.

	Before matching			After matching		
	HRT	G-HRT	P	HRT	G-HRT	P
Patients	2083	1482		1133	1133	
Age (y)	31.25±4.9	33.33±5.55	<.0001	31.72±4.8	31.79±4.88	0.7285
Days of embryo freezing	322.35±470.33	359.91±470.26	0.0188	331.71±453.18	362.12±462.17	0.114
BMI (kg/m2)	21.56±2.6	21.92±2.61	<.0001	21.58±2.37	21.76±2.49	0.0683
Previous conception (%)	1266 (60.78)	1029 (69.43)	<.0001	703 (62.05)	729 (64.34)	0.2574
Scar uterus (%)	358 (17.19)	320 (21.59)	0.001	188 (16.59)	207 (18.27)	0.2928
Infertility factors						
Tubal factors (%)	1616 (77.58)	1164 (78.54)	0.4945	910 (80.32)	894 (78.91)	0.4041
Ovulation obstacle (%)	115 (5.52)	66 (4.45)	0.1525	69 (6.09)	55 (4.85)	0.196
Male factor (%)	551 (26.45)	404 (27.26)	0.5912	274 (24.18)	290 (25.6)	0.4369
No. of embryos transferred (%)			0.0003			0.6568
1	660 (31.69)	555 (37.45)		377 (33.27)	387 (34.16)	
2	1423 (68.31)	927 (62.55)		756 (66.73)	746 (65.84)	
Phase of embryo transferred (%)			<.0001			0.4994
Cleavage embryo	1037 (49.78)	639 (43.12)		504 (44.48)	520 (45.9)	
Blastocyst	1046 (50.22)	843 (56.88)		629 (55.52)	613 (54.1)	

**Table 3:** Baseline characteristics of patients with multiple failures of embryo implantation.

	HRT	G-HRT	P-value
Patients	302	785	
Age (y)	33.28±5.13	33.82±5.47	0.1416
Days of embryo freezing	465.09±493.11	446.68±486.12	0.5777
BMI (kg/m2)	21.42±2.05	21.58±2.44	0.2974
Previous conception (%)	520 (45.9)	527 (67.13)	0.2373
Scar uterus (%)	62 (20.53)	155 (19.75)	0.7719
Infertility factors			
Tubal factors (%)	247 (81.79)	629 (80.13)	0.5352
Ovulation obstacle (%)	21 (6.95)	43 (5.48)	0.3545
Male factor (%)	69 (22.85)	178 (22.68)	0.9515
No. of embryos transferred (%)			0.195
1	113 (37.42)	261 (33.25)	
2	189 (62.58)	524 (66.75)	
Phase of embryo transferred (%)			0.1336
Cleavage embryo	98 (32.45)	293 (37.32)	
Blastocyst	204 (67.55)	492 (62.68)	

Live birth and secondary outcomes are presented in **Table 4**. The live birth rate in the G-HRT group was significantly higher than that of HRT group for women with multiple failure of embryo implantation (HRT: 38.74% [117/302], G-HRT: 45.48% [357/785], P=0.0449); the same was for biochemical pregnancy (HRT: 58.28% [176/302], G-HRT: 67.13% [527/785], P=0.0062) and clinical pregnancy (HRT: 48.68% [147/302], G-HRT: 55.92% [439/785], P=0.0318). However, the live birth was similar between two groups for women with no/one failure of embryo implantation. In addition, the implantation rate of G-HRT group was higher than that of HRT group for women with on failure, and the biochemical pregnancy and clinical pregnancy of G-HRT group was higher than that of HRT group for women with one failure. A significantly higher endometrial thickness was seen in the G-HRT group than the HRT group in all the three sub-studies (No failure, HRT: 9.3 ± 1.66 mm, G-HRT: 9.69 ± 2.09 mm, P<.0001; One failure, HRT: 9.14 ± 1.54 mm, G-HRT: 9.69 ± 2.02 mm, P<.0001; Multiple failure, HRT: 9.15 ± 1.54 mm, G-HRT: 9.68 ± 1.95 mm, P<.0001).

**Table 4:** Clinical outcomes stratified by times of embryo implantation failures.

	No failure of embryo implantation			One failure of embryo implantation			Multiple failure of embryo implantation		
	HRT	G-HRT	P-value	HRT	G-HRT	P-value	HRT	G-HRT	P-value
Patients	1165	1165		1133	1133		302	785	
Endometrial thickness (mm)	9.3±1.66	9.69±2.09	<.0001	9.14±1.54	9.69±2.02	<.0001	9.15±1.54	9.68±1.95	<.0001
Biochemical pregnancy (%)	714 (61.29)	753 (64.64)	0.0943	726 (64.08)	779 (68.76)	0.0184	176 (58.28)	527 (67.13)	0.0062
Clinical pregnancy (%)	610 (52.36)	651 (55.88)	0.0883	637 (56.22)	687 (60.64)	0.0331	147 (48.68)	439 (55.92)	0.0318
Implantation rate (%)	743 (37.75)	830 (41.62)	0.0128	830 (43.94)	879 (46.78)	0.0798	190 (38.7)	560 (42.78)	0.1175
Pregnancy loss (%)	96 (15.74)	117 (17.97)	0.2899	94 (14.76)	111 (16.16)	0.4815	28 (19.05)	81 (18.45)	0.8722
Live birth (%)	498 (42.75)	527 (45.24)	0.2261	535 (47.22)	570 (50.31)	0.1413	117 (38.74)	357 (45.48)	0.0449

**Figure 2:**
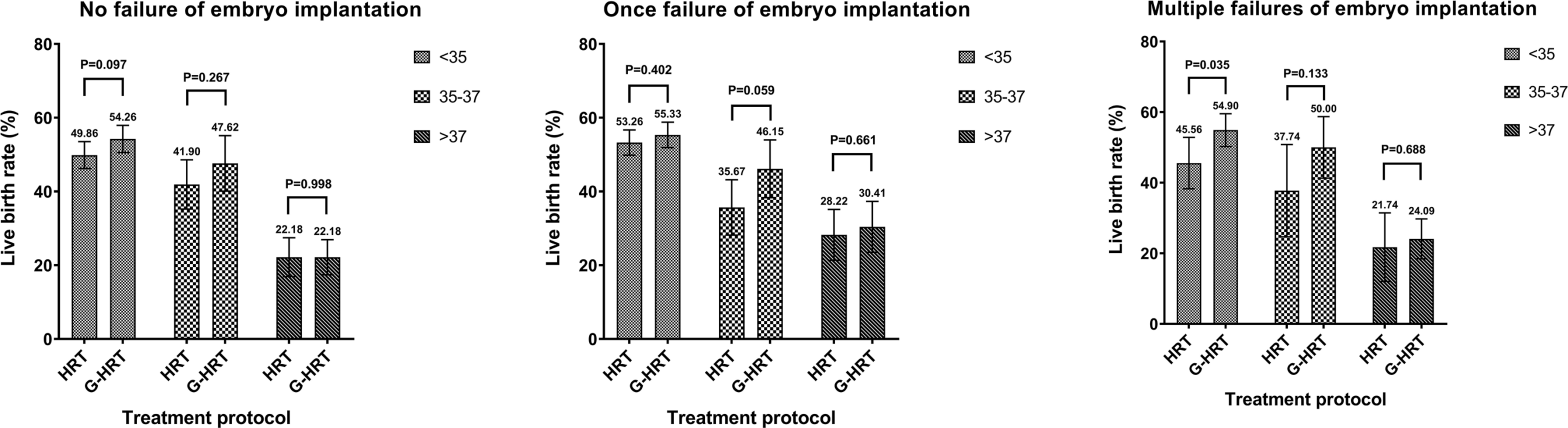
Live birth rate stratified by times of embryo implantation failures and age.

When the participants were stratified by age, the trend of a higher live birth rate was found among women aged less than 38 in G-HRT group and the difference was inconspicuous if women’s age exceeded 37 years (**Figure 2**). The multivariate logistic regression GEE model showed that GnRH-a pretreatment among women with multiple failures resulted in a higher live birth rate with an adjust odds ratio of 1.50 (95% confidence interval [CI], 1.12 to 2.00; P=0.0067) (**Table 5**), while the efficacy of GnRH-a pretreatment was not significant in women with no/one failures.

**Table 5:** Multivariate logistic regression GEE model with odds ratios for live birth.

Independent covariates	No history of embryo implantation failure		Once history of embryo implantation failure		Multiple history of embryo implantation failure	
	Adjusted Odds ratio (95% CI)	P-value	Adjusted Odds ratio (95% CI)	P-value	Adjusted Odds ratio (95% CI)	P-value
Treatment protocol (G-HRT vs. HRT)	1.18 (1.00,1.41)	0.0542	1.18 (0.99,1.40)	0.0659	1.50 (1.12,2.00)	0.0067
Age						
<35 years (reference)						
35–37 years	0.77 (0.61,0.97)	0.0294	0.68 (0.53,0.89)	0.0039	0.90 (0.62,1.28)	0.5476
>37 years	0.29 (0.23,0.37)	<.0001	0.46 (0.35,0.60)	<.0001	0.39 (0.27,0.55)	<.0001
BMI						
<18.5 (reference)						
18.5-24	1.04 (0.77,1.41)	0.7898	1.05 (0.77,1.44)	0.7541	1.06 (0.69,1.64)	0.7891
>24	1.11 (0.78,1.58)	0.5658	1.08 (0.74,1.57)	0.7014	0.97 (0.56,1.67)	0.9061
Previous conception (yes vs. no)	1.02 (0.84,1.25)	0.8399	0.88 (0.73,1.06)	0.1869	0.97 (0.72,1.31)	0.8454
Scar uterus (yes vs. no)	0.76 (0.60,0.95)	0.0155	0.96 (0.76,1.22)	0.7504	0.85 (0.60,1.19)	0.3366
Tubal factors (yes vs. no)	1.07 (0.87,1.30)	0.5365	1.10 (0.88,1.37)	0.3960	1.33 (0.94,1.88)	0.1055
Ovulation obstacle (yes vs. no)	1.13 (0.79,1.61)	0.5022	1.22 (0.85,1.76)	0.2863	1.11 (0.65,1.88)	0.7099
No. of embryos transferred (2 vs. 1)	1.94 (1.49,2.51)	<.0001	1.83 (1.48,2.26)	<.0001	2.26 (1.69,3.01)	<.0001
Phase of embryo transferred (blastocyst vs. cleavage embryo)	2.46 (1.90,3.18)	<.0001	2.21 (1.80,2.72)	<.0001	3.00 (2.24,4.03)	<.0001

## Discussion

This study analyzed the efficacy of the GnRH-a administration before HRT for FET in women with different times of embryo implantation failures. The results show that GnRH-a pretreatment is beneficial to raise the live birth rate for women with multiple failures (>1 embryo transfers) of embryo implantation. GnRH-a pretreatment do not increase the live birth rate in women with no/one failure of embryo implantation. In addition, GnRH-a pretreatment can improve endometrial thickness on progesterone initiation day independently from the numbers of failed embryo implantations.

How to improve the success rate of FETs has attracted more and more attention from reproductive medicine specialists as the proportion of FETs increased. This study was the first one to compare the efficacy of GnRH-a pretreatment before HRT for FET in women with different times of embryo implantation failures. To our knowledge, this retrospective study of 9263 samples is the largest analysis of comparison between HRT and G-HRT. We used the PSM method to control for the potential confounders in no/one failure of embryo implantation sub-studies. However, we did not match the two groups for women with multiple failures because there was no significant difference in basic information. The PSM method is useful for observational studies in which treatment allocation is non-random and can be viewed as an approach seeking to replicate random assignment in conventional randomized controlled trials (11). Observational studies of ART are unique from other studies because of the presence of multiple treatment cycles per women may have several cycles which will lead to clustering effect. Therefore, GEE model was used instead of conventional logistic regression for multivariate analysis.

Our study has some limitations. It can be found that the basic conditions before PSM in the G-HRT group are worse than those in the HRT group. The main reason is that doctors tend to choose G-HRT protocol for difficult patients, such as having only one precious embryo, multiple failures of implantation and thin endometrium in previous cycles. Although the confounding factors were balanced by the PSM and multivariate logistic regression GEE model, some slight differences could not be reflected in the data. Therefore, we hold that the results of this study are conservative and the difference between G-HRT group and HRT group may actually be more obvious. A large prospective cohort study or randomized controlled study is urgently needed for more accurate comparisons. This study has another limitation. Because the preimplantation genetic testing has not been widely used in our center, the selection of embryos was mainly based on morphological grading. Therefore, we could not exclude the confounding effects caused by embryo aneuploidy, which should be taken into full consideration in further studies when exploring the effect of GnRH-a pretreatment on endometrial receptivity.

The efficacy of GnRH-a pretreatment before HRT has been controversial. Most randomized trials have shown that there is no difference in clinical pregnancy rates between HRT and G-HRT (4, 12–14). In addition, GnRH agonist might pose more financial burden, prolong treatment time and the women may suffer from menopausal symptoms resulting from hypo-estrogenic state. Therefore, relevant reviews do not recommend using GnRH agonist before HRT (5). However, many studies have shown that long-term GnRH-a administration in infertile women with endometriosis or adenomyosis can improves endometrial receptivity and significantly increases the chances of pregnancy (7, 15). This suggests that the treatment protocol should vary from person to person.

Most women undergoing assisted reproductive technology treatment would experience one or more transfer failures. The cause of failure can be summarized as gamete/embryo factors and uterine/endometrial factors. When multiple high-quality embryos failed to be implanted, uterine/endometrial factors were considered as the main reason of multiple failures (9). Our motivation for this study was to speculate that more failures of embryo implantation were associated with lower endometrial receptivity. In order to explore the independent relationship between the times of embryo implantation failures and endometrial receptivity, we excluded all other infertility that may affect endometrial receptivity, such as endometriosis, polycystic ovary syndrome and endometritis. Presently, the evidence of GnRH agonist pretreatment for women with RIF is limited. Yang et al. (16) reported that pituitary suppression before frozen embryo transfer is beneficial for patients suffering from idiopathic repeated implantation failure. A RCT with a small sample, however, showed no significant differences in pregnancy rates between HRT and G-HRT (17).

It needs to be explained that the women with multiple failures of embryo implantation in this study were different from women with RIF. The definition of RIF has not yet been unified, more commonly used definitions include: (1) failure to achieve a clinical pregnancy after >3 embryo transfers with high quality embryos or the transfer of ≥10 embryos in multiple transfers (18); (2) failure to achieve a clinical pregnancy after transfer of at least four good-quality embryos in a minimum of three fresh or frozen cycles in a woman under the age of 40 years (9). However, the criteria for women with multiple failures (>1 embryo transfers) are much less strict than most definition of RIF. The results of the study are meaningful, because multiple failures cover a much larger population than RIF.

In our study, the G-HRT group had a thicker endometrium than that of HRT group. This conclusion is consistent with previous studies (12, 19). GnRH-a pretreatment can also get a thicker endometrium in fresh-embryo transfer cycles, besides in the FET cycles (6, 20). Endometrium thickness has been used as a marker of the uterine receptivity to embryos, and as a predictor of IVF-ET success (21, 22). Although related mechanisms are still unclear, it is likely to be associated with the hypothesis of endometrial recovery. A break of constant menstrual cycling caused by prolonged pituitary down-regulation may restore full function to the steroid-sensitive systems (23). The wider implications of this outcome is that GnRH-a pretreatment may be suitable for women with thin endometrium.

Age is a key factor affecting endometrial receptivity and embryo quality. According to the results of age stratification of one failure of embryo implantation sub-group, the pretreatment of GnRH-a can obtain greater benefits in the 35-37 age group (HRT: 35.67%, G-HRT: 46.15%, P=0.059), followed by the younger (<35 years old) group (HRT: 53.26%, G-HRT: 55.33%, P=0.402), while older (>37 years old) women do not benefit significantly (HRT: 28.22%, G-HRT: 30.41%, P=0.661). The possible explanation for this parabolic phenomenon is that most young women do not have endometrial problems, and the efficacy of GnRH-a pretreatment and is limited (24, 25). Similarly, the main reason of implantation failure is the high rate of embryo aneuploidy for older women, which coincides with most RIF’s definition of restricting the age of women (9). However, the clinic outcomes of G-HRT group were numerically higher than HRT group at all ages for multiple failures of embryo implantation sub-group, which suggested GnRH-a pretreatment is beneficial at all ages for multiple failures women (<35 years old, HRT: 45.56%, G-HRT: 54.90%, P=0.035; 35-37 years old, HRT: 37.74%, G-HRT: 50.00%, P=0.133; >37 years old, HRT: 21.74%, G-HRT: 24.09%, P=0.699). Only a superficial subgroup analysis was performed in this study, so these speculations still require special research and further discussion.

## Conclusions

This retrospective study supports the hypothesis that long-acting GnRH agonist administration in the early follicular phase before hormonal replacement treatment for frozen-thawed embryo transfer can effectively improve the clinical outcomes of women with at least two failures of embryo implantation. For women with no/one failure of embryo implantation, the effect of GnRH-a pretreatment was not obvious. Due to the limitations of retrospective studies, this conclusion needs to be confirmed by prospective studies. Furthermore, our study found that GnRH-a pretreatment can significantly increase the endometrial thickness on progesterone initiation day independently from the numbers of failed embryo implantations, which prompt GnRH-a pretreatment might be beneficial for women with thin endometrium.

## Data Availability Statement

The raw data supporting the conclusions of this article will be made available by the authors, without undue reservation.

## Ethics Statement

The studies involving human participants were reviewed and approved by Ethics committee: Reproductive Medicine Ethics Committee of Jiangxi Maternal and Child Health Hospital; Affiliation name: Jiangxi Maternal and Child Health Hospital. Written informed consent for participation was not required for this study in accordance with the national legislation and the institutional requirements.

## Author Contributions

LX: conception of the idea, study design, data analysis and drafting of the manuscript. LT: study design, interpretation of data analysis results and revising of the manuscript. SZ and JH: revising of the manuscript. QW: guidance on the research design, revising of the manuscript and final approval of the version to be published. All authors contributed to the article and approved the submitted version.

## Conflict of Interest

The authors declare that the research was conducted in the absence of any commercial or financial relationships that could be construed as a potential conflict of interest.

## Publisher’s Note

All claims expressed in this article are solely those of the authors and do not necessarily represent those of their affiliated organizations, or those of the publisher, the editors and the reviewers. Any product that may be evaluated in this article, or claim that may be made by its manufacturer, is not guaranteed or endorsed by the publisher.

## References

[1] Shapiro BS Daneshmand ST Garner FC Aguirre M Hudson C. Clinical Rationale for Cryopreservation of Entire Embryo Cohorts in Lieu of Fresh Transfer. Fertil Steril (2014) 102:3–9. doi: 10.1016/j.fertnstert.2014.04.018 24842675

[2] Madani T Ramezanali F Yahyaei A Hasani F Bagheri Lankarani N Mohammadi Yeganeh L. Live Birth Rates After Different Endometrial Preparation Methods in Frozen Cleavage-Stage Embryo Transfer Cycles: A Randomized Controlled Trial. Arch Gynecol Obstet (2019) 299:1185–91. doi: 10.1007/s00404-019-05062-7 30707360

[3] Roque M Haahr T Geber S Esteves SC Humaidan P. Fresh Versus Elective Frozen Embryo Transfer in IVF/ICSI Cycles: A Systematic Review and Meta-Analysis of Reproductive Outcomes. Hum Reprod Update (2019) 25:2–14. doi: 10.1093/humupd/dmy033 30388233

[4] Dal Prato L Borini A Cattoli M Bonu MA Sciajno R Flamigni C. Endometrial Preparation for Frozen-Thawed Embryo Transfer With or Without Pretreatment With Gonadotropin-Releasing Hormone Agonist. Fertil Steril (2002) 77:956–60. doi: 10.1016/S0015-0282(02)02960-6 12009350

[5] Ghobara T Gelbaya TA Ayeleke RO. Cycle Regimens for Frozen-Thawed Embryo Transfer. Cochrane Database Syst Rev (2017) 7:CD003414. doi: 10.1002/14651858.CD003414.pub3 28675921 PMC6483463

[6] Surrey ES Silverberg KM Surrey MW Schoolcraft WB. Effect of Prolonged Gonadotropin-Releasing Hormone Agonist Therapy on the Outcome of *In Vitro* Fertilization-Embryo Transfer in Patients With Endometriosis. Fertil Steril (2002) 78:699–704. doi: 10.1016/S0015-0282(02)03373-3 12372443

[7] Niu Z Chen Q Sun Y Feng Y. Long-Term Pituitary Downregulation Before Frozen Embryo Transfer Could Improve Pregnancy Outcomes in Women With Adenomyosis. Gynecol Endocrinol (2013) 29:1026–30. doi: 10.3109/09513590.2013.824960 24006906

[8] Xu B Geerts D Hu S Yue J Li Z Zhu G. The Depot GnRH Agonist Protocol Improves the Live Birth Rate Per Fresh Embryo Transfer Cycle, But Not the Cumulative Live Birth Rate in Normal Responders: A Randomized Controlled Trial and Molecular Mechanism Study. Hum Reprod (2020) 35:1306–18. doi: 10.1093/humrep/deaa086 32478400

[9] Coughlan C Ledger W Wang Q Liu F Demirol A Gurgan T. Recurrent Implantation Failure: Definition and Management. Reprod BioMed Online (2014) 28:14–38. doi: 10.1016/j.rbmo.2013.08.011 24269084

[10] Dodge LE Farland LV Correia KFB Missmer SA Seidler EA Wilkinson J. Choice of Statistical Model in Observational Studies of ART. Hum Reprod (2020) 35:1499–504. doi: 10.1093/humrep/deaa050 PMC7368396 32424400

[11] Whittaker W Anselmi L Kristensen SR Lau YS Bailey S Bower P. Associations Between Extending Access to Primary Care and Emergency Department Visits: A Difference-In-Differences Analysis. PloS Med (2016) 13:e1002113. doi: 10.1371/journal.pmed.1002113 27598248 PMC5012704

[12] Simon A Hurwitz A Zentner BS Bdolah Y Laufer N. Transfer of Frozen-Thawed Embryos in Artificially Prepared Cycles With and Without Prior Gonadotrophin-Releasing Hormone Agonist Suppression: A Prospective Randomized Study. Hum Reprod (1998) 13:2712–7. doi: 10.1093/humrep/13.10.2712 9804219

[13] Azimi Nekoo E Chamani M Shahrokh Tehrani E Hossein Rashidi B Davari Tanha F Kalantari V. Artificial Endometrial Preparation for Frozen-Thawed Embryo Transfer With or Without Pretreatment With Depot Gonadotropin Releasing Hormone Agonist in Women With Regular Menses. J Family Reprod Health (2015) 9:1–4. 25904960 PMC4405510

[14] Davar R Eftekhar M Tayebi N. Transfer of Cryopreserved- Thawed Embryos in a Cycle Using Exogenous Steroids With or Without Prior Gonadotrophin-Releasing Hormone Agonist. J Med Sci (2007) 7(5):880–3. doi: 10.3923/jms.2007.880.883

[15] Park CW Choi MH Yang KM Song IO. Pregnancy Rate in Women With Adenomyosis Undergoing Fresh or Frozen Embryo Transfer Cycles Following Gonadotropin-Releasing Hormone Agonist Treatment. Clin Exp Reprod Med (2016) 43:169–73. doi: 10.5653/cerm.2016.43.3.169 PMC5039310 27689040

[16] Yang X Huang R Wang YF Liang XY. Pituitary Suppression Before Frozen Embryo Transfer Is Beneficial for Patients Suffering From Idiopathic Repeated Implantation Failure. J Huazhong Univ Sci Technol Med Sci (2016) 36:127–31. doi: 10.1007/s11596-016-1554-2 26838753

[17] Davar R Dashti S Omidi M. Endometrial Preparation Using Gonadotropin-Releasing Hormone Agonist Prior to Frozen-Thawed Embryo Transfer in Women With Repeated Implantation Failure: An RCT. Int J Reprod BioMed (2020) 18:319–26. doi: 10.18502/ijrm.v13i5.7150 PMC7306065 32637860

[18] Thornhill AR deDie-Smulders CE Geraedts JP Harper JC Harton GL Lavery SA. ESHRE PGD Consortium ‘Best Practice Guidelines for Clinical Preimplantation Genetic Diagnosis (PGD) and Preimplantation Genetic Screening (PGS)’. Hum Reprod (2005) 20:35–48. doi: 10.1093/humrep/deh579 15539444

[19] Qi Q Luo J Wang Y Xie Q. Effects of Artificial Cycles With and Without Gonadotropin-Releasing Hormone Agonist Pretreatment on Frozen Embryo Transfer Outcomes. J Int Med Res (2020) 48:300060520918474. doi: 10.1177/0300060520918474 32586174 PMC7432982

[20] Ren J Sha A Han D Li P Geng J Ma C. Does Prolonged Pituitary Down-Regulation With Gonadotropin-Releasing Hormone Agonist Improve the Live-Birth Rate in *In Vitro* Fertilization Treatment? Fertil Steril (2014) 102:75–81. doi: 10.1016/j.fertnstert.2014.03.030 24746740

[21] Al-Ghamdi A Coskun S Al-Hassan S Al-Rejjal R Awartani K. The Correlation Between Endometrial Thickness and Outcome of *In Vitro* Fertilization and Embryo Transfer (IVF-ET) Outcome. Reprod Biol Endocrinol (2008) 6:37. doi: 10.1186/1477-7827-6-37 18764940 PMC2543019

[22] Richter KS Bugge KR Bromer JG Levy MJ. Relationship Between Endometrial Thickness and Embryo Implantation, Based on 1,294 Cycles of *In Vitro* Fertilization With Transfer of Two Blastocyst-Stage Embryos. Fertil Steril (2007) 87:53–9. doi: 10.1016/j.fertnstert.2006.05.064 17081537

[23] Edwards RG. Clinical Approaches to Increasing Uterine Receptivity During Human Implantation. Hum Reprod (1995) 2:60–6. doi: 10.1093/humrep/10.suppl_2.60 8745302

[24] Yaron Y Botchan A Amit A Kogosowski A Yovel I Lessing JB. Endometrial Receptivity: The Age-Related Decline in Pregnancy Rates and the Effect of Ovarian Function. Fertil Steril (1993) 60:314–8. doi: 10.1016/S0015-0282(16)56104-4 8339830

[25] Wang L Lv S Mao W Bai E Yang X. Fecundity Disorders in Older Women: Declines in Follicular Development and Endometrial Receptivity. BMC Womens Health (2020) 20:115. doi: 10.1186/s12905-020-00979-7 32487204 PMC7268486

